# 4-Carb­oxy-2-methyl-1*H*-imidazol-3-ium-5-carboxyl­ate monohydrate

**DOI:** 10.1107/S1600536808040221

**Published:** 2008-12-06

**Authors:** Yu-Ping Guo

**Affiliations:** a School of Pharmacy, Jiangxi Science and Technology Normal University, Jiangxi 330013, People’s Republic of China

## Abstract

In the title compound, C_6_H_6_N_2_O_4_·H_2_O, one carboxyl group is deprotonated and one imidazole N atom is protonated. The organic mol­ecule, excluding methyl H atoms, is essentially planar, with an r.m.s. deviation of 0.0156 (1) Å. In the crystal structure, inter­molecular N—H⋯O hydrogen bonds link mol­ecules into chains along the *b* axis; these chains are further linked *via* O—H⋯O hydrogen bonds involving the water O atoms and carboxyl O atoms, generating a two-dimensional supra­molecular framework.

## Related literature

For details of related structures, see: Sun *et al.* (2006 ▶); Nie *et al.* (2007 ▶). For applications as functional materials, see: Liang *et al.* (2002 ▶); Qin *et al.* (2002 ▶); Li *et al.* (1998 ▶). For biological activities, see: Ucucu *et al.* (2001 ▶); Maeda *et al.* (1984 ▶); Quattara *et al.* (1987 ▶); Seko *et al.* (1991 ▶). For the synthesis of the title compound, see: Anderson *et al.* (1989 ▶).
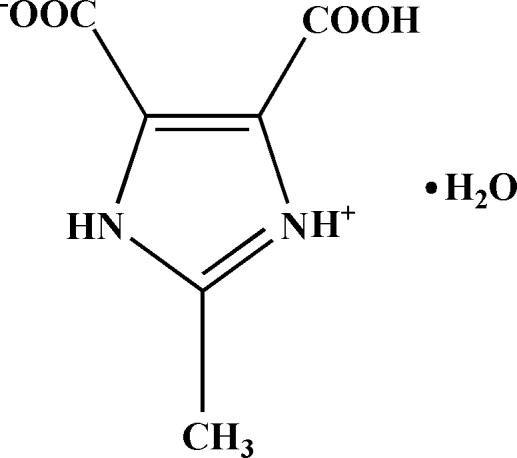


## Experimental

### 

#### Crystal data

C_6_H_6_N_2_O_4_·H_2_O
*M* *_r_* = 188.14
Monoclinic, 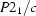

*a* = 8.491 (2) Å
*b* = 14.280 (4) Å
*c* = 6.5385 (17) Å
β = 97.386 (5)°
*V* = 786.2 (4) Å^3^
*Z* = 4
Mo *K*α radiation
μ = 0.14 mm^−1^
*T* = 295 (2) K
0.23 × 0.09 × 0.08 mm

#### Data collection

Bruker SMART APEX area-detector diffractometer
Absorption correction: multi-scan (*SADABS*; Sheldrick, 1996 ▶) *T*_min_ = 0.969, *T*_max_ = 0.995
4506 measured reflections
1538 independent reflections
1246 reflections with *I* > 2σ(*I*)
*R*_int_ = 0.032

#### Refinement

*R*[*F* ^2^ > 2σ(*F* ^2^)] = 0.056
*wR*(*F* ^2^) = 0.131
*S* = 1.05
1538 reflections
128 parameters
4 restraints
H atoms treated by a mixture of independent and constrained refinement
Δρ_max_ = 0.25 e Å^−3^
Δρ_min_ = −0.26 e Å^−3^

### 

Data collection: *SMART* (Bruker, 2002 ▶); cell refinement: *SAINT* (Bruker, 2002 ▶); data reduction: *SAINT*; program(s) used to solve structure: *SHELXS97* (Sheldrick, 2008 ▶); program(s) used to refine structure: *SHELXL97* (Sheldrick, 2008 ▶); molecular graphics: *SHELXTL* (Sheldrick, 2008 ▶); software used to prepare material for publication: *SHELXTL*.

## Supplementary Material

Crystal structure: contains datablocks I, global. DOI: 10.1107/S1600536808040221/wn2295sup1.cif

Structure factors: contains datablocks I. DOI: 10.1107/S1600536808040221/wn2295Isup2.hkl

Additional supplementary materials:  crystallographic information; 3D view; checkCIF report

## Figures and Tables

**Table 1:** Hydrogen-bond geometry (Å, °)

*D*—H⋯*A*	*D*—H	H⋯*A*	*D*⋯*A*	*D*—H⋯*A*
O1*W*—H1*WB*⋯O4^i^	0.85 (1)	2.05 (1)	2.887 (3)	168 (4)
O1*W*—H1*WA*⋯O3^ii^	0.85 (1)	2.00 (1)	2.839 (3)	173 (4)
N2—H2⋯O1^iii^	0.86	1.86	2.716 (3)	176
N1—H1⋯O1*W*	0.86	1.83	2.689 (3)	177
O3—H3⋯O2	0.86 (1)	1.59 (2)	2.447 (2)	179 (3)

Symmetry codes: (i) 
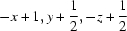
; (ii) 
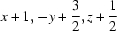
; (iii) 
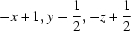
.

**Fig. 1.**
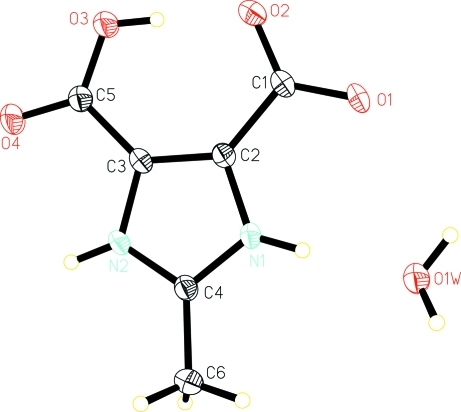
The molecular structure of the title compound, with displacement ellipsoids drawn at the 30% probability level. Hydrogen atoms are drawn as spheres of arbitrary radius.

**Fig. 2.**
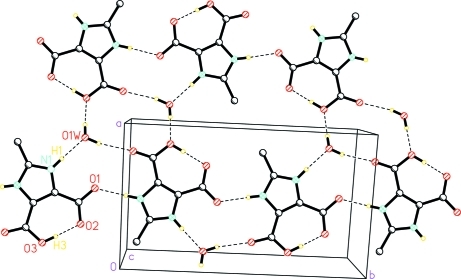
Packing diagram of the title structure, showing the intermolecular N—H···O and O—H···O hydrogen bonds as dashed lines. Methyl H atoms are omitted for clarity.

## supplementary crystallographic information

### Comment

In recent years, N-heterocyclic carboxylic acids have attracted considerable interest as ligands in metal complexes because of their structural diversity (Nie *et al.*, 2007; Sun *et al.*) and their potential applications as functional materials (Liang *et al.*, 2002; Qin *et al.*, 2002; Li *et al.*, 1998). Sun *et al.* (2006) have prepared the inner salt, 4-carboxy-2-(pyridinium-4-yl)-1*H*-imidazole-5-carboxylate monohydrate; in its crystal structure, one carboxyl group is deprotonated and the pyridyl group is protonated. Nie *et al.* (2007) have reported the mononuclear complex, diaquabis(5-carboxy-2-methyl-1*H*-imidazole-4-carboxylate- *κ*^2^*N*^3^,*O*^4^)cadmium(II). Imidazole derivatives have a wide range of biological activities such as analgesic (Ucucu *et al.*, 2001), anti-inflammatory (Maeda *et al.*, 1984), antiparasitic (Quattara *et al.*, 1987), antiepileptic and platelet aggregation inhibitors (Seko *et al.*, 1991). I report here the crystal structure of 4-carboxy-2-methyl-1*H*-3-imidazolium-5-carboxylate monohydrate.

As shown in Fig. 1, the asymmetric unit consists of a neutral C_6_H_6_N_2_O_4_ molecule and one water molecule. The organic molecule, excluding methyl hydrogen atoms, is essentially planar, with an r.m.s. deviation of 0.0156 (1) Å. The C1-containing carboxylate group forms an intramolecular hydrogen bond with the neighboring C5-containing carboxyl group.

In the crystal structure, intermolecular N—H···O hydrogen bonds link the molecules into chains along the *b* axis; these chains are further linked *via* O—H···O hydrogen bonds involving the water O atoms and carboxyl O atoms, generating a two-dimensional supramolecular framework (Fig. 2).

### Experimental

The title compound was synthesized according to a revised procedure (Anderson *et al.*, 1989). 2-Methylimidazole (3.0 g) was added to a mixture of concentrated sulfuric acid (40 ml) and water (30 ml) at 363 K. This was followed by the careful addition of powdered potassium dichromate (22 g). After 30 min the mixture was poured into ice-cold water. The white precipitates were collected by filtration, and washed with water. Recrystallization from hot water afforded colorless block crystals of the title compound. Yield: 1.8 g (44%).

### Refinement

The carboxyl and water H atoms were located in a difference Fourier map and refined with *U*_iso_(H) = 1.5*U*_eq_(O). The O—H distances of the water molecule were restrained to 0.85 (1) Å; however, that of the carboxyl was refined freely. All other H-atoms were positioned geometrically and refined using a riding model with C—H (methyl) = 0.96 Å, N—H = 0.86 Å; *U*_iso_(H) = k*U*_eq_(carrier atom), where k = 1.2 for N and 1.5 for C.

### Crystal data

**Table:**

C_6_H_6_N_2_O_4_·H_2_O	*F*(000) = 392
*M**_r_* = 188.14	*D*_x_ = 1.589 Mg m^−^^3^
Monoclinic, *P*2_1_/*c*	Mo *K*α radiation, λ = 0.71073 Å
Hall symbol: -P 2ybc	Cell parameters from 927 reflections
*a* = 8.491 (2) Å	θ = 2.4–24.3°
*b* = 14.280 (4) Å	µ = 0.14 mm^−^^1^
*c* = 6.5385 (17) Å	*T* = 295 K
β = 97.386 (5)°	Block, colorless
*V* = 786.2 (4) Å^3^	0.23 × 0.09 × 0.08 mm
*Z* = 4	

### Data collection

**Table:**

Bruker SMART APEX area-detector diffractometer	1538 independent reflections
Radiation source: fine-focus sealed tube	1246 reflections with *I* > 2σ(*I*)
graphite	*R*_int_ = 0.032
φ and ω scans	θ_max_ = 26.0°, θ_min_ = 2.4°
Absorption correction: multi-scan (*SADABS*; Sheldrick, 1996)	*h* = −8→10
*T*_min_ = 0.969, *T*_max_ = 0.995	*k* = −17→17
4506 measured reflections	*l* = −8→7

### Refinement

**Table:**

Refinement on *F*^2^	Primary atom site location: structure-invariant direct methods
Least-squares matrix: full	Secondary atom site location: difference Fourier map
*R*[*F*^2^ > 2σ(*F*^2^)] = 0.056	Hydrogen site location: inferred from neighbouring sites
*wR*(*F*^2^) = 0.131	H atoms treated by a mixture of independent and constrained refinement
*S* = 1.05	*w* = 1/[σ^2^(*F*_o_^2^) + (0.0559*P*)^2^ + 0.4711*P*] where *P* = (*F*_o_^2^ + 2*F*_c_^2^)/3
1538 reflections	(Δ/σ)_max_ < 0.001
128 parameters	Δρ_max_ = 0.25 e Å^−^^3^
4 restraints	Δρ_min_ = −0.26 e Å^−^^3^

### Special details

**Table:**

Geometry. All e.s.d.'s (except the e.s.d. in the dihedral angle between two l.s. planes) are estimated using the full covariance matrix. The cell e.s.d.'s are taken into account individually in the estimation of e.s.d.'s in distances, angles and torsion angles; correlations between e.s.d.'s in cell parameters are only used when they are defined by crystal symmetry. An approximate (isotropic) treatment of cell e.s.d.'s is used for estimating e.s.d.'s involving l.s. planes.
Refinement. Refinement of *F*^2^ against ALL reflections. The weighted *R*-factor *wR* and goodness of fit *S* are based on *F*^2^, conventional *R*-factors *R* are based on *F*, with *F* set to zero for negative *F*^2^. The threshold expression of *F*^2^ > σ(*F*^2^) is used only for calculating *R*-factors(gt) *etc*. and is not relevant to the choice of reflections for refinement. *R*-factors based on *F*^2^ are statistically about twice as large as those based on *F*, and *R*- factors based on ALL data will be even larger.

### Fractional atomic coordinates and isotropic or equivalent isotropic displacement parameters (Å^2^)

**Table:**

	*x*	*y*	*z*	*U*_iso_*/*U*_eq_	
O1	0.5312 (2)	0.88207 (11)	0.2430 (3)	0.0404 (5)	
O3	0.1439 (2)	0.68027 (12)	0.1429 (3)	0.0430 (5)	
H3	0.191 (3)	0.7337 (12)	0.157 (5)	0.065*	
N2	0.5293 (2)	0.56877 (13)	0.2531 (3)	0.0287 (5)	
H2	0.5126	0.5094	0.2501	0.034*	
O2	0.2822 (2)	0.83103 (12)	0.1822 (3)	0.0436 (5)	
N1	0.6490 (2)	0.70121 (13)	0.2840 (3)	0.0284 (5)	
H1	0.7232	0.7424	0.3045	0.034*	
C2	0.4901 (3)	0.72074 (15)	0.2375 (3)	0.0265 (5)	
C3	0.4139 (3)	0.63632 (15)	0.2178 (3)	0.0265 (5)	
C1	0.4314 (3)	0.81906 (15)	0.2198 (4)	0.0303 (6)	
C4	0.6708 (3)	0.60933 (16)	0.2927 (4)	0.0283 (5)	
C5	0.2437 (3)	0.61108 (17)	0.1680 (4)	0.0335 (6)	
O4	0.2045 (2)	0.52952 (12)	0.1540 (3)	0.0506 (6)	
C6	0.8249 (3)	0.56070 (18)	0.3429 (4)	0.0405 (7)	
H6A	0.8557	0.5333	0.2199	0.061*	
H6B	0.8148	0.5124	0.4425	0.061*	
H6C	0.9043	0.6049	0.3987	0.061*	
O1W	0.8762 (2)	0.83332 (13)	0.3359 (4)	0.0563 (6)	
H1WA	0.961 (3)	0.827 (2)	0.419 (5)	0.084*	
H1WB	0.840 (4)	0.8884 (12)	0.345 (5)	0.084*	

### Atomic displacement parameters (Å^2^)

**Table:**

	*U*^11^	*U*^22^	*U*^33^	*U*^12^	*U*^13^	*U*^23^
O1	0.0417 (11)	0.0175 (8)	0.0613 (13)	−0.0014 (7)	0.0041 (9)	−0.0002 (8)
O3	0.0293 (10)	0.0278 (10)	0.0697 (14)	−0.0004 (7)	−0.0020 (9)	0.0000 (9)
N2	0.0313 (11)	0.0172 (9)	0.0371 (12)	0.0002 (8)	0.0025 (9)	−0.0009 (8)
O2	0.0360 (11)	0.0272 (9)	0.0664 (14)	0.0062 (8)	0.0025 (9)	0.0008 (8)
N1	0.0287 (11)	0.0206 (10)	0.0351 (11)	−0.0044 (8)	0.0016 (8)	−0.0009 (8)
C2	0.0288 (12)	0.0229 (11)	0.0275 (13)	0.0001 (9)	0.0028 (10)	−0.0002 (9)
C3	0.0330 (13)	0.0200 (11)	0.0267 (12)	0.0015 (9)	0.0042 (10)	0.0005 (9)
C1	0.0380 (14)	0.0214 (12)	0.0315 (14)	0.0013 (10)	0.0046 (10)	0.0005 (9)
C4	0.0304 (13)	0.0230 (12)	0.0311 (13)	−0.0008 (9)	0.0028 (10)	−0.0005 (9)
C5	0.0307 (13)	0.0278 (14)	0.0413 (15)	−0.0016 (10)	0.0023 (11)	0.0009 (10)
O4	0.0399 (11)	0.0261 (10)	0.0835 (16)	−0.0082 (8)	−0.0013 (10)	−0.0008 (9)
C6	0.0346 (14)	0.0309 (13)	0.0551 (18)	0.0050 (11)	0.0019 (12)	0.0018 (12)
O1W	0.0395 (12)	0.0292 (10)	0.0942 (18)	−0.0035 (8)	−0.0148 (11)	0.0009 (11)

### Geometric parameters (Å, °)

**Table:**

O1—C1	1.232 (3)	C2—C3	1.367 (3)
O3—C5	1.299 (3)	C2—C1	1.489 (3)
O3—H3	0.861 (10)	C3—C5	1.484 (3)
N2—C4	1.329 (3)	C4—C6	1.481 (3)
N2—C3	1.373 (3)	C5—O4	1.212 (3)
N2—H2	0.8600	C6—H6A	0.9600
O2—C1	1.271 (3)	C6—H6B	0.9600
N1—C4	1.325 (3)	C6—H6C	0.9600
N1—C2	1.373 (3)	O1W—H1WA	0.849 (10)
N1—H1	0.8600	O1W—H1WB	0.850 (10)
			
C5—O3—H3	112 (2)	O2—C1—C2	117.2 (2)
C4—N2—C3	109.54 (19)	N1—C4—N2	107.8 (2)
C4—N2—H2	125.2	N1—C4—C6	126.1 (2)
C3—N2—H2	125.2	N2—C4—C6	126.2 (2)
C4—N1—C2	109.81 (19)	O4—C5—O3	123.6 (2)
C4—N1—H1	125.1	O4—C5—C3	120.0 (2)
C2—N1—H1	125.1	O3—C5—C3	116.4 (2)
C3—C2—N1	106.37 (19)	C4—C6—H6A	109.5
C3—C2—C1	132.4 (2)	C4—C6—H6B	109.5
N1—C2—C1	121.2 (2)	H6A—C6—H6B	109.5
C2—C3—N2	106.5 (2)	C4—C6—H6C	109.5
C2—C3—C5	132.1 (2)	H6A—C6—H6C	109.5
N2—C3—C5	121.3 (2)	H6B—C6—H6C	109.5
O1—C1—O2	125.4 (2)	H1WA—O1W—H1WB	110 (2)
O1—C1—C2	117.4 (2)		
			
C4—N1—C2—C3	0.1 (3)	C3—C2—C1—O2	1.2 (4)
C4—N1—C2—C1	179.5 (2)	N1—C2—C1—O2	−178.0 (2)
N1—C2—C3—N2	0.0 (2)	C2—N1—C4—N2	−0.1 (3)
C1—C2—C3—N2	−179.3 (2)	C2—N1—C4—C6	−178.7 (2)
N1—C2—C3—C5	−179.6 (2)	C3—N2—C4—N1	0.1 (3)
C1—C2—C3—C5	1.1 (4)	C3—N2—C4—C6	178.7 (2)
C4—N2—C3—C2	−0.1 (3)	C2—C3—C5—O4	178.1 (3)
C4—N2—C3—C5	179.6 (2)	N2—C3—C5—O4	−1.4 (4)
C3—C2—C1—O1	−178.9 (2)	C2—C3—C5—O3	−2.3 (4)
N1—C2—C1—O1	1.9 (3)	N2—C3—C5—O3	178.2 (2)

### Hydrogen-bond geometry (Å, °)

**Table:**

*D*—H···*A*	*D*—H	H···*A*	*D*···*A*	*D*—H···*A*
O1W—H1WB···O4^i^	0.85 (1)	2.05 (1)	2.887 (3)	168 (4)
O1W—H1WA···O3^ii^	0.85 (1)	2.00 (1)	2.839 (3)	173 (4)
N2—H2···O1^iii^	0.86	1.86	2.716 (3)	176
N1—H1···O1W	0.86	1.83	2.689 (3)	177
O3—H3···O2	0.86 (1)	1.59 (2)	2.447 (2)	179 (3)

Symmetry codes: (i) −*x*+1, *y*+1/2, −*z*+1/2; (ii) *x*+1, −*y*+3/2, *z*+1/2; (iii) −*x*+1, *y*−1/2, −*z*+1/2.
